# CRISPR/Cas9 revolutionizes *Macleaya cordata* breeding: a leap in sanguinarine biosynthesis

**DOI:** 10.1093/hr/uhae024

**Published:** 2024-01-16

**Authors:** Mengshan Sun, Xiaohong Zhong, Li Zhou, Wei Liu, Rong Song, Peng Huang, Jianguo Zeng

**Affiliations:** Hunan Key Laboratory of Traditional Chinese Veterinary Medicine, Hunan Agricultural University, Changsha 410128, Hunan, China; Hunan Institute of Agricultural Environment and Ecology, Hunan Academy of Agricultural Sciences, Changsha 410125, Hunan, China; College of Horticulture, Hunan Agricultural University, Changsha 410128, Hunan, China; College of Horticulture, Hunan Agricultural University, Changsha 410128, Hunan, China; Hunan Institute of Agricultural Environment and Ecology, Hunan Academy of Agricultural Sciences, Changsha 410125, Hunan, China; Hunan Key Laboratory of Traditional Chinese Veterinary Medicine, Hunan Agricultural University, Changsha 410128, Hunan, China; College of Veterinary Medicine, Hunan Agricultural University, Changsha 410128, Hunan, China; Hunan Institute of Agricultural Environment and Ecology, Hunan Academy of Agricultural Sciences, Changsha 410125, Hunan, China; College of Animal Science and Technology, Hunan Agricultural University, Changsha 410128, Hunan, China; Hunan Key Laboratory of Traditional Chinese Veterinary Medicine, Hunan Agricultural University, Changsha 410128, Hunan, China; College of Veterinary Medicine, Hunan Agricultural University, Changsha 410128, Hunan, China; National and Local Union Engineering Research Center of Veterinary Herbal Medicine Resource and Initiative, Hunan Agricultural University, Changsha 410128, Hunan, China

Dear Editor,

For many years, the agricultural industry has relied heavily on the use of antibiotics as growth promoters (AGPs) in animal feeding practices. Recognizing the associated health risks, the EU, the USA, and China have banned AGPs, prompting global research into sustainable alternatives for animal growth and health [1]. Sanguinarine, an important growth promoter used in feed for its antibacterial and growth-promoting effects, is produced in abundance by *Macleaya cordata* [2]. With the increasing demand for sanguinarine, it is necessary to accelerate breeding research on *M. cordata* with the goal of increasing the sanguinarine content. In recent years, precise gene-editing technologies such as CRISPR/Cas9 have offered novel approaches for overcoming the limitations of traditional breeding, with notable success in medicinal plants such as *Dioscorea* spp. [3] and *Cannabis sativa* [4]. These examples serve as pioneering forays in the field of genetic manipulation of medicinal plants and demonstrate the potential of using CRISPR/Cas9 to make targeted genetic changes [2]. Here we report a successful CRISPR/Cas9-mediated genome editing system in *M. cordata* and applied this system to successfully knock out branches in the sanguinarine synthesis pathway to obtain mutant plants with significantly increased sanguinarine levels.

Initially, we searched the entire length of the sequences and CDS regions of the *McPDS* gene from the *M. cordata* genome and transcript information. The *PDS* gene is involved in converting phytoene to carotene and the carotenoid precursor phytofluene. It is usually used for proofing genome editing as a visible marker in plants since the destruction of its function results in albinism [3]. According to the draft genome sequence, *McPDS* is a single gene that spans ~14 kb and consists of 13 exons. For *McPDS* editing, we selected a 20-bp sequence (5′-GAAACAATGAAATGCTTACA-3′) (Fig. 1a) in an exon of the gene as the spacer sequence using the CRISPR-P 2.0 tool (http://crispr.hzau.edu.cn/CRISPR2/). The spacer sequence was subsequently cloned and inserted into the CRISPR vector pRGEB32 to obtain the recombinant plasmid pRGEB32-PDS (Fig. 1b). The recombinant plasmid was subsequently transformed into *Agrobacterium tumefaciens* strain GV3101, which was subsequently used to infect *M. cordata* leaves and stems. *McPDS* transformants were screened on antibiotic selection medium supplemented with hygromycin (10 μg/ml). A total of 68 positive transgenic plantlets were ultimately obtained. Among them, 36 were completely albino, while three were chimeras exhibiting mosaic albino phenotypes (Fig. 1c). Genomic DNA was prepared from an albino plantlet using the primers PDS-F and PDS-R (Fig. 1a) to amplify the target site region, which was subsequently identified by sequencing. Multiple peaks after the cut sites indicated mutations in the albino phenotype plantlet (Fig. 1d).

**Figure 1:**
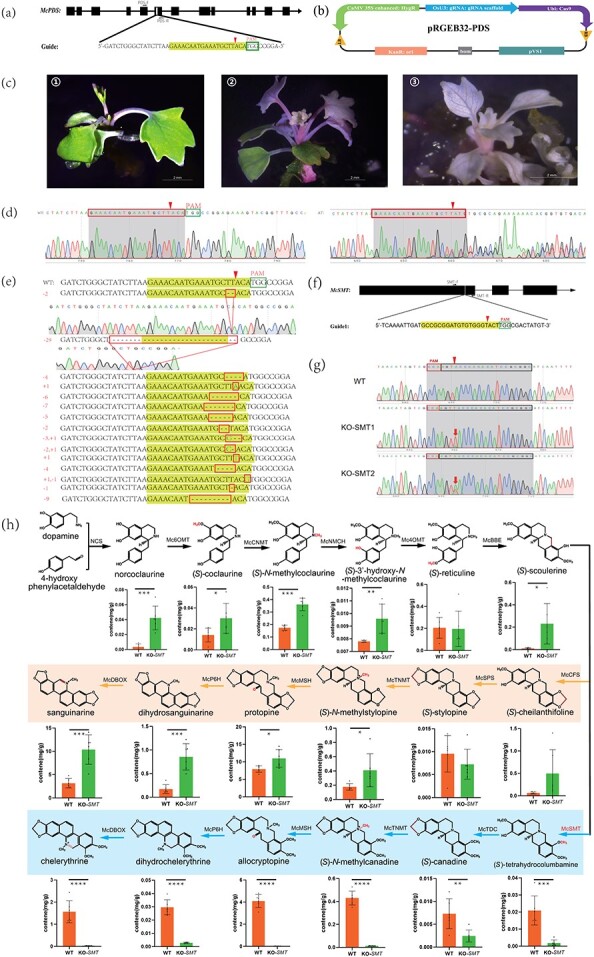
CRISPR/Cas9-based genome editing of *M. cordata* and the levels of isoquinoline alkaloid synthesis pathway compounds in *McSMT* gene knockout plants. **a** Gene structure of gRNA target sites. The black bars represent exons and the connecting lines indicate introns. The PAM sequences are surrounded by green boxes, the gRNA sequences are shaded in yellow, and the red arrows indicate the sites cut by the Cas9 protein. **b** Schematic representation of the targeting vector used to generate the *McPDS* genome-edited events. The OsU3 promoter drives the expression of the sgRNA, the Ubi promoter drives Cas9, and the CaMV35S promoter drives the expression of the hygromycin resistance gene as a selectable marker. **c** Phenotypes of CRIPSR/Cas9-induced mutant *M. cordata*. Photographs 1–3 show a wild-type plantlet (1), a typical variegated transformant with chimeric mutations (2), and an albino transformant (3). Scale bar: 2 mm. **d** Sequencing chromatograms of wild-type (WT) plantlets (left) and albino-type (AT) plantlets (right). The PAM sequences are surrounded by green boxes, the gRNA sequences are surrounded by red boxes, and the red arrows indicate the sites cut by the Cas9 protein. The multiple peaks after the cut sites indicate the presence of mutations. **e** Mutations around the gRNA target sites of the *McPDS* gene. The gRNA sequences are shaded in yellow, the red boxes indicate mutations, deleted nucleotides are shown as black dashes, and inserted nucleotides are shown in green. **f** Gene structure of the gRNA target sites. The black bars represent exons and the connecting lines indicate introns. The PAM sequences are surrounded by green boxes, the gRNA sequences are shaded in yellow, and the red arrows indicate the sites cut by the Cas9 protein. **g***McSMT* gene mutation and gene mutation map. WT represents the target site sequence of the *McSMT* gene in wild-type plants. KO-SMT1 represents *McSMT* gene mutation type 1, KO-SMT2 represents *McSMT* gene mutation type 2, and the red arrow indicates the mutated nucleotide site. Both alleles are knockout mutations, with one base (T) insertion in one allele and a different base (A) insertion in the other. **h** Content of isoquinoline alkaloid synthesis pathway compounds in *McSMT* gene knockout and WT *M. cordata* plants. The orange shadow represents the sanguinarine branch pathway, and the blue shadow represents the chelerythrine branch pathway.

To assess the types of mutations in the *McPDS* gene more precisely, DNA from 68 positive transgenic plants was amplified via PCR with the primers PDS-F and PDS-R. The mutation types were determined by cloning the PCR products and sequencing. We screened and sequenced 678 clones from the DNA of 68 positive transgenic plantlets, and 15 mutation types were identified (Fig. 1e). The mutations included insertions, deletions, and substitutions in the vicinity of the Cas9 cleavage site. Deletions were the most predominant, the longest being 29 bases. No mutations were detected in 183 clones; all others contained mutations. Among the remaining clones, the -2 mutation (TT) was the most common type, found in 247 clones, followed by the -29 mutation type, found in 78 clones; there were relatively few clones with other mutation types. Among the 68 transgenic-positive seedlings, no mutation of the *McPDS* gene was found in 9 seedlings, 8 were homozygous, 23 were bi-allelic, 16 were heterozygous, and 12 were chimeric (Table 1). Mutations at the designated target site were detected in 59 of the 68 transgenic-positive seedlings, indicating that the *McPDS* gene targeting efficiency in *M. cordata* was as high as 86.76%, which was much greater than that in soybean [5] (43.4–48.1%) and close to that in rice [6] (85.4%). Off-target gene editing is a major concern in plants [7–9]. Hence, we searched for potential off-target sites in the *M. cordata* genome database and selected five sites with 15- to 18-bp sequences matching the target sequence that also had a PAM (NGG) sequence near the site. Ten positive plants were randomly selected for PCR analysis and sequencing. All potential off-target sites were confirmed by sequencing to be free of mutations.

**Table 1:** Mutation types and bleaching phenotype on 68 transgenic positive seedlings.

**Mutation type**	**Number of seedlings**	**Bleaching phenotype**
No mutations	9	Green
Homozygous	8	Completely albino
Bi-allelic	23	Completely albino
Heterozygous	16	Green
Chimeric	5	Completely albino
	4	Green
	3	Mosaic albino

After the construction of the system, we knocked out the *McSMT* gene, which is a branch of the sanguinarine synthesis pathway; no other family members were found by whole-genome sequencing. The target location and sequence are shown in Fig. 1f (5′-GCCGCGGATGTGTGGGTACT-3′). A total of 12 regenerated plants were obtained by genetic transformation, and the growth performance of these plants was consistent with that of the wild-type plants. When the height of the aboveground part of the plant reached 6–8 cm, one leaf was cut from each plant for total DNA extraction, and the positive transgenic plants were identified via PCR (SMT-F/SMT-R) and sequencing. All 12 plants were found to be transgenic. The target site fragment was amplified, and the base mutation at the target site was identified by sequencing. The results showed that there were two types of single-base insertion mutations at the target site of the *McSMT* gene (Fig. 1g); WT in the figure represents the wild-type without mutation. The two single-base insertions were found, a T and an A. The sequencing peaks of 12 positive plants were all single-base nested peaks of mutation sites, indicating that all the mutant plants were heterozygous. We speculate that these 12 plants are likely to have differentiated and developed from the same mutant material, so they have the same mutation type. The off-target detection results showed that the agent had no off-target effects.

UPLC–QQQ–MS was used to quantitatively analyze the content of chelerythrine branch pathway compounds in whole plants (including roots, stems, and leaves), i.e. six *McSMT* gene knockout mutant plants and six wild-type plants, and the content of each compound was calculated according to the peak area and standard curve (Fig. 1h). Overall, compared with wild-type plants, (*S*)-scoulerine could not be effectively catalyzed to produce (*S*)-tetrahydrocolumbamine after *McSMT* knockout, which directly led to significant reductions of (*S*)-canadine, (*S*)-*N*-methylcanadine, allocryptopine, dihydrochelerythrine, chelerythrine, and other compounds downstream of the pathway to extremely low levels. This finding indicated that the chelerythrine pathway was effectively truncated. The contents of compounds such as norcoclaurine, (*S*)-coclaurine, (*S*)-*N*-methylcoclaurine, (*S*)-3′-hydroxy-*N*-methylcoclaurine, and (*S*)-scoulerine before branching significantly increased. The contents of (*S*)-cheilanthifoline, (*S*)-*N*-methylstylopine, protopine, dihydrosanguinarine, and sanguinarine compounds in the sanguinarine branch pathway were significantly increased, especially those of dihydrosanguinarine and sanguinarine. The average content increased 4.95- and 3.29-fold, respectively. This finding was similar to results found in other plants [7, 10].

The expression of key genes in the alkaloid synthesis pathway was analyzed via qRT–PCR and transcriptomic analysis. The results showed that the relative expression of the *McSMT* gene was lower in the mutant plants. The expression levels of the remaining genes were higher. In addition, a total of 30 differentially expressed transcription factors were identified, among which the MYB family was the most represented. MYB3, MYB5, MYB82, and bHLH130 were identified as the four transcription factors that are most likely to play a regulatory role in the metabolism of benzylisoquinoline alkaloids in *M. cordata*.

In summary, this study successfully constructed a CRISPR/Cas9-mediated gene-editing system for *M. cordata*. On this basis, plants with significantly increased sanguinarine content were obtained, which will become important material for the subsequent breeding of *M. cordata*.

## Acknowledgements

This work was supported by Hunan Innovative Province Construction Special Fund Grant, Yuelu Mountain Seed Industry Innovation Project (2021NK1012) and the Hunan Provincial Natural Science Foundation of China (2023JJ40367, 2023JJ30341, 2021JJ20032).

## Author contributions

M.S., P.H., and J.Z. conceived and designed the study. M.S. and P.H. wrote the manuscript. M.S. and L.Z. conducted the experimental work. R.S., X.Z., and W.L. participated in the results interpretation. All authors read and approved the final manuscript.

## Data availability statement

Two genes from *M. cordata*, *McPDS* (GenBank accession number OR502428) and *McSMT* (OR502429), have been deposited in GenBank. Transcriptome raw data in this study have been deposited in the SRA database (https://www.ncbi.nlm.nih.gov/sra) under project number PRJNA1018289.

## Conflict of interest

The authors declare no competing interests.
